# Hepatic Metabolomics Investigation in Acute and Chronic Murine Toxoplasmosis

**DOI:** 10.3389/fcimb.2018.00189

**Published:** 2018-06-05

**Authors:** Xiao-Qing Chen, Hany M. Elsheikha, Rui-Si Hu, Gui-Xue Hu, Shu-Ling Guo, Chun-Xue Zhou, Xing-Quan Zhu

**Affiliations:** ^1^State Key Laboratory of Veterinary Etiological Biology, Key Laboratory of Veterinary Parasitology of Gansu Province, Lanzhou Veterinary Research Institute, Chinese Academy of Agricultural Sciences, Lanzhou, China; ^2^College of Animal Science and Technology, Jilin Agricultural University, Changchun, China; ^3^Faculty of Medicine and Health Sciences, School of Veterinary Medicine and Science, University of Nottingham, Loughborough, United Kingdom; ^4^Department of Parasitology, Shandong University School of Basic Medicine, Jinan, China

**Keywords:** toxoplasmosis, host-pathogen interaction, liver, non-targeted metabolomics, LC-MS/MS

## Abstract

*Toxoplasma gondii* poses a great threat to human health, with no approved vaccine available for the treatment of *T. gondii* infection. *T. gondii* infections are not limited to the brain, and may also affect other organs especially the liver. Identification of host liver molecules or pathways involved in *T. gondii* replication process may lead to the discovery of novel anti-*T. gondii* targets. Here, we analyzed the metabolic profile of the liver of mice on 11 and 30 days postinfection (dpi) with type II *T. gondii* Pru strain. Global metabolomics using liquid chromatography-tandem mass spectrometry (LC-MS/MS) identified 389 significant metabolites from acutely infected mice; and 368 from chronically infected mice, when compared with control mice. Multivariate statistical analysis revealed distinct metabolic signatures from acutely infected, chronically infected and control mice. Infection influenced several metabolic processes, in particular those for lipids and amino acids. Metabolic pathways, such as steroid hormone biosynthesis, primary bile acid biosynthesis, bile secretion, and biosynthesis of unsaturated fatty acids were perturbed during the whole infection process, particularly during the acute stage of infection. The present results provide insight into hepatic metabolic changes that occur in BALB/c mice during acute and chronic *T. gondii* infection.

## Introduction

*Toxoplasma gondii* infection poses a serious health problem worldwide (Dubey, 2008; Elsheikha, 2008; Dupont et al., 2012). This parasite has a complex life cycle that involves both sexual and asexual phases. Sexual reproduction results in the formation and shedding of the oocysts by the felid definitive host (Sullivan and Jeffers, 2012). Ingestion of food or drinking water contaminated with oocysts is one of the most important sources of infection (Dubey and Beattie, 1988). Asexual reproduction results in the formation of tachyzoites and bradyzoites-containing tissue cysts, marking acute and chronic infection, respectively (Munoz et al., 2011; Sullivan and Jeffers, 2012). The transformation of tachyzoites into tissue cysts is immune-mediated and leads to the establishment of latent infection (Dubey and Frenkel, 1976). Ingesting undercooked or raw meat that contains tissue cysts is another important source of infection (Dubey and Beattie, 1988). Although the brain and spinal cord are the primarly affected organs (Elsheikha and Zhu, 2016), *T. gondii* infections are not limited to the central nervous system, and may also affect other organs. Several hepatic abnormalities, including hepatomegaly, hepatitis, granuloma, necrosis, cholestatic jaundice, cirrosis, and abnormalities in liver enzymes have been observed in the context of *T. gondii* infection (Vischer et al., 1967; Weitberg et al., 1979; Wendum et al., 2002). However, the definite pathophysiological links between *T. gondii* and extraneural manifestations are yet to be established.

The association between *T. gondii* infection and hepatic injury has attracted considerable attention due to the significant roles played by the liver in maintaining many body functions. The mechanisms leading to hepato-pathologic disturbances in *T. gondii*-infected mice have been investigated using transcriptomics (He et al., 2016a) and proteomics (He et al., 2016b). However, the mechanisms underlying the hepatic pathological outcomes of *T. gondii* infection remain unuclear. *T. gondii*, a strict intracellular parasite, relies on host cells to obtain its energetic and nutritional needs (Kafsack and Llinas, 2010). The parasite utilizes essential nutrients from the host cell, including tryptophan (Pfefferkorn, 1984; Dai et al., 1994; Naemat et al., 2016); arginine (Fox et al., 2004); polyamines (Seabra et al., 2004); purines (Schwartzman and Pfefferkorn, 1982; Chaudhary et al., 2004); cholesterol (Coppens et al., 2000; Coppens and Joiner, 2003) and other nutrients (Dimier and Bout, 1998; Charron and Sibley, 2002). Hence, it is reasonable to assume that infected cells display different metabolic phenotypes, and that the metabolomic alterations and associated dysregulation of biochemical pathways may underlie the hepatopathy observed in toxoplasmosis.

In recent years, transcriptomics and proteomics approaches have been employed to study the interaction between *T. gondii* and its host (Nelson et al., 2008; Pittman et al., 2014; He et al., 2016a,b; Cong et al., 2017). Metabolomics is a rapidly emerging field of “omics” research and can provide a global analysis of the metabolic changes in biological systems in response to external stimuli or perturbation (Nicholson et al., 1999; Yang et al., 2006; Holmes et al., 2008). Metabolomics has been as a powerful method to profile metabolic alterations associated with microbial infections (Antunes et al., 2011; Shin et al., 2011; Nguyen et al., 2015). In previous studies, we characterized the metabolomic profiles of the brain (Zhou et al., 2015), serum (Zhou et al., 2016), and spleen (Chen et al., 2017) of mice infected with *T. gondii*. In this study, we extended our metabolomics analysis to include the liver due to its important role in maintaining many metabolic processes in the body. Here, the liver metabolic profile was compared between mice with acute infection and mice with chronic infection using liquid chromatography-tandem mass spectrometry (LC-MS/MS). This approach revealed metabolic differences between infected and uninfected mice, and between acutely infected and chronically infected mice.

## Materials and methods

### Ethical approval

All experiments were approved by the Animal Administration and Ethics Committee of Lanzhou Veterinary Research Institute, Chinese Academy of Agricultural Sciences (Permit No. LVRIAEC2016-007). Mice were handled in strict accordance with good laboratory animal practice according to the Animal Ethics Procedures and Guidelines of the People's Republic of China. All efforts were made to minimize animal suffering and to minimize the number of animals used in the experiments.

### Mice infection

Thirty-nine, 6-week-old, female, BALB/c mice were obtained from the Laboratory Animal Center of Lanzhou Veterinary Research Institute, Chinese Academy of Agricultural Sciences. Mice were housed in microisolator cages under specific-pathogen free (SPF) conditions, with controlled temperature (22 ± 2°C) and 12 h light/dark cycles, and were given water and standard food pellets *ad libitum*. Mice were randomly allocated into three groups: acutely infected group (*n* = 13), chronically infected group (*n* = 13) and healthy uninfected group (*n* = 13). Each mouse in the infected group was infected orally with 10 tissue cysts of *T. gondii* type II Pru strain that were isolated from mice brain 40 days postinfection (dpi) in 100 μl phosphate buffered saline solution (PBS). Control mice were gavaged with 100 μl PBS only. All mice were observed daily for the development of clinical signs and for survival throughout the entire experiment. Also, weight loss was monitored as a sign of morbidity.

### Collection of tissues and characterization of infection

At 11 days postinfection (dpi), 10 mice from the acutely infected group were sacrificed and their livers were collected. A further 10 mice representing chronically infected group, and 10 uninfected controls, were sacrificed and their livers were collected at 30 dpi. Mice were anesthetized by isoflurane inhalation and then sacrificed by exsanguination via cardiac puncture. Livers were rapidly removed, cut into small pieces, rinsed with saline solution (0.9% NaCl w/v), snap-frozen in liquid nitrogen, and stored at −80°C. Mice brain collected at 30 dpi were checked for the presence of *T. gondii* cysts using a light microscope. Other organs (including heart, lung, muscle, small intestine and kidney) were examined for the presence of *T. gondii*, as previously described (Hill et al., 2006). Briefly, total genomic DNA was extracted from these various tissues using QIAamp® DNA Mini kit in accordance with the manufacturer's instructions (QIAGEN, Germany). Isolated DNA was then used as a template for PCR amplification of the *T. gondii* B1 gene using the specific primers (5′-AACGGG CGA GTA GCA CCT GAG GAG-3′ and 5′-TGG GTC TAC GTC GAT GGC ATG ACA AC-3′). Positive control (DNA from *T. gondii*) and negative (deionized water) control samples were included in each PCR run. Sections of livers from 9 mice (3 from each of the acutely infected, chronically infected, and control groups) were collected and processed for routine histopathological examination. Tissues were fixed by submersion in 10% neutral buffered formalin for 1 week, then dehydrated in a graded series of ethanol and embedded in paraffin wax. Sections were made (5 μm) using a microtome, then stained with hematoxylin-eosin (H&E) and examined under an optical microscope (Olympus, Tokyo, Japan).

### Extraction of metabolites

Liver samples were taken from storage in a −80°C freezer and allowed to thaw at 4°C. The organic protein precipitation method was used to extract metabolites. Briefly, 25 mg tissue samples were weighed and then pulverized in liquid nitrogen. Then, 800 μl methanol/water (1:1) solution and 3 mm (mean diameter) steel beads were added to each sample. Using a TissueLyser bead-mill homogenizer (QIAGEN, Hilden, Germany), samples were homogenized via vibrating at 60 Hz for 5 min. Subsequently, samples were centrifuged at 25,000 g for 10 min at 4°C. To evaluate the reproducibility and stability of the LC-MS/MS system, 200 μl supernatant of each sample was mixed to generate a pooled quality control (QC) sample. The supernatant from each sample was separated and freeze dried. Finally, dried supernatant samples were reconstituted in deionized water and analyzed by mass spectrometry.

### LC-MS/MS analysis

An ultra-performance liquid chromatography (UPLC) system (Waters, USA) was used to perform all analyses. The Chromatographic separation was achieved using an ACQUITY UPLC BEH C18 column (100 mm ^*^ 2.1 mm, 1.7 μm, Waters, USA) at a column temperature of 50°C and flow rate of 0.4 ml/min, wherein the mobile phase consisted of solvent A (water + 0.1% formic acid) and solvent B (acetonitrile + 0.1% formic acid). The following gradient elution conditions were used to elute metabolites: 100% phase A for 0-2 min; 0% to 100% phase B for ~11 min; 100% phase B for 11–13 min; 100% phase A for 13-15 min. Injection volume per sample was 10 μl. The metabolites eluted from the column were detected using a high-resolution tandem mass spectrometer SYNAPT G2 XS QTOF (Waters, USA) in positive and negative electrospray ionization modes. For the positive ionization mode, the capillary voltage and the cone voltage were set at 2 kV and 40 V, respectively. For the negative ionization mode, these parameters were set at 1 kV and 40 V, respectively. Centroid MSE mode was used to obtain the mass spectrometry data. The primary scan ranged from 50 Da to 1200 Da with a scanning time of 0.2 s. All the parent ionizations were fragmented at 20-40 V and the information from the fragments was collected. During data acquisition, the LE signal was gained every 3 s for real-time quality correction. Furthermore, quality control samples (10 samples) were collected in order to evaluate the stability of the instrument during measurements.

### Data processing and statistical analysis

UPLC-MS/MS raw data files were imported into Progenesis QI software (Waters, UK). Data were processed for possible adducts, peak alignment, peak detection, deconvolution, dataset filtering, noise reduction, compound identification, and normalization with sum method. Chromatograms were aligned to a reference QC run and selected adducts included [M+H]^+^, [M+Na]^+^, [M+K]^+^, [M+NH4]^+^ for ESI^+^ and [M−H]^−^, [M+Cl]^−^ for ESI– with 2.5 AU filter. Peak picking was set to exclude ions eluted before 0.5 min and after 9 min at 1% and 92% organic solution, respectively. The raw data were normalized using a global scaling factor to align all injections to a reference run by adjusting for the quantitative abundance of each detectable feature, correcting for experimental variation based on deviation from the median. The results were exported in.CSV format for further analysis and reporting of selected ions by their accurate mass and retention time pair. The normalized peak data were further processed by an in-house software metaX (Wen et al., 2017). Those features that were detected in less than 50% of QC samples or 80% of biological samples were removed, and the remaining peaks with missing values were imputed with the k-nearest neighbor algorithm to further improve the data quality. Then, the ions that demonstrated relative standard deviation (RSD) greater than 30% in QC pools were filtered out.

Multivariate statistical analyses including Principal Component Analysis (PCA) and Partial Least Squares-Discriminant Analysis (PLS-DA) were performed to discriminate infected mice from control mice. PCA was performed for outlier detection and batch effects evaluation using the pre-processed dataset. Supervised PLS-DA was conducted through metaX to discriminate the different variables between groups. The condition of *p*-values obtained from a two-tailed, Student's *t*-test on the normalized peak areas (*q-*values) ≤ 0.05 and Fold Change (FC) ≥1.2 or ≤ 0.833, were taken into consideration during the selection of candidate metabolites. Log2 FC based on metabolite abundance was used to demonstrate how the differential abundance of liver metabolites varied between the mouse groups. Differential metabolites were identified based on *q*-values (adjusted *p*-values using Benjamini-Hochberg procedure) and false discovery rate (FDR) of ≤ 0.05, FC ≥ 1.2 or ≤ 0.833, and variable importance in projection (VIP) score ≥ 1 from the PLS-DA model. Data were log2-transformed prior to cluster analysis and the generation of heat-maps using R, to show distinctions in the metabolic state between infected mice and controls, and between infected mice at different stages of infection.

### Identification of metabolites

Metabolite identification was performed by matching the exact molecular mass data (*m*/*z*) of samples with the online KEGG (www.genome.jp/kegg/) database. A mass error range of 10 ppm was set to match the compound and obtain a mass accuracy score (Zhong et al., 2017). Progenesis QI software was used to match the theoretical isotope distribution in the database, and obtain the isotopic similarity score of the metabolites. Then, all the possible compounds corresponding to each parent ion in the first-order spectrum were theoretically fragmented according to their molecular structure to obtain the theoretical fragment ions. The spectra of all theoretical fragment ions together formed the theoretical secondary spectrum. Then, we set the mass error range of 10 ppm, and matched the actual secondary spectrum of the experimental samples with the theoretical secondary spectrum. Scores of the matching similarity between the spectra were counted. Finally, the three scores were combined into total assessment scores, which were used to screen the metabolites.

## Results

### Clinical and histopathological analysis

**Figure 1:**
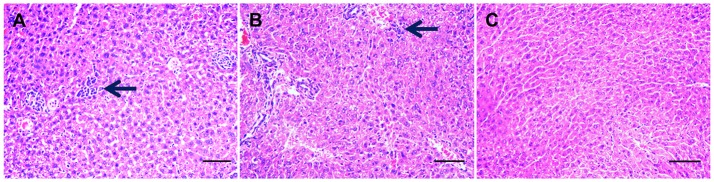
Histopathological changes in the liver tissues from *Toxoplasma gondii*-infected mice. Histopathological analysis was conducted on livers from mock- and *T. gondii*-infected mice at days 11 and 30 postinfection. Photomicrographs of liver tissue sections stained with hematoxylin and eosin (original magnification, × 100). **(A)** Liver section from a mouse on day 11 postinfection. Liver shows multifocal mononuclear cell aggregations (mainly lymphocytes, arrow) with numerous vacuolated hepatocyte. **(B)** At 30 dpi, liver shows mild mononuclear cellular infiltration (arrow), increased numbers of Von Kupffer cells besides numerous pyknotic hepatocytes. **(C)** Hepatic tissue from mock-infected, control mice shows a normal histological structure.

Infected mice exhibited a time-dependent increase in progression of disease. At 7 dpi, infected mice developed mild loss of appetite and ruffled hair coat. On day 11 postinfection, clinical signs had progressed to those typically observed during acute infection, including anorexia, weight loss, edema and ruffled hair. By day 14 postinfection infected mice began to recover from acute signs, and by day 30 postinfection all infected mice had restored their normal physical state and developed chronic infection. As expected, control mice did not show any clinical manifestations throughout the experiment. PCR results of all examined tissues indicated that all infected mice tested positive, but control mice tested negative. A histopathological study of liver tissue from all mice was also performed, in order to ascertain both acute (11 dpi) and chronic (30 dpi) histopathologies. Notable histological changes were observed in acutely infected mice, which showed multifocal mononuclear cell aggregations with numerous vacuolated hepatocytes (Figure 1A). These changes featured less prominently in chronically infected mice (Figure 1B). Livers of the control mice showed no gross histopathological abnormalities (Figure 1C). The original image of Figures 1A–C are Figures S1–S3, respectively.

### Metabolite profiling of liver during *T. gondii* infection

**Figure 2:**
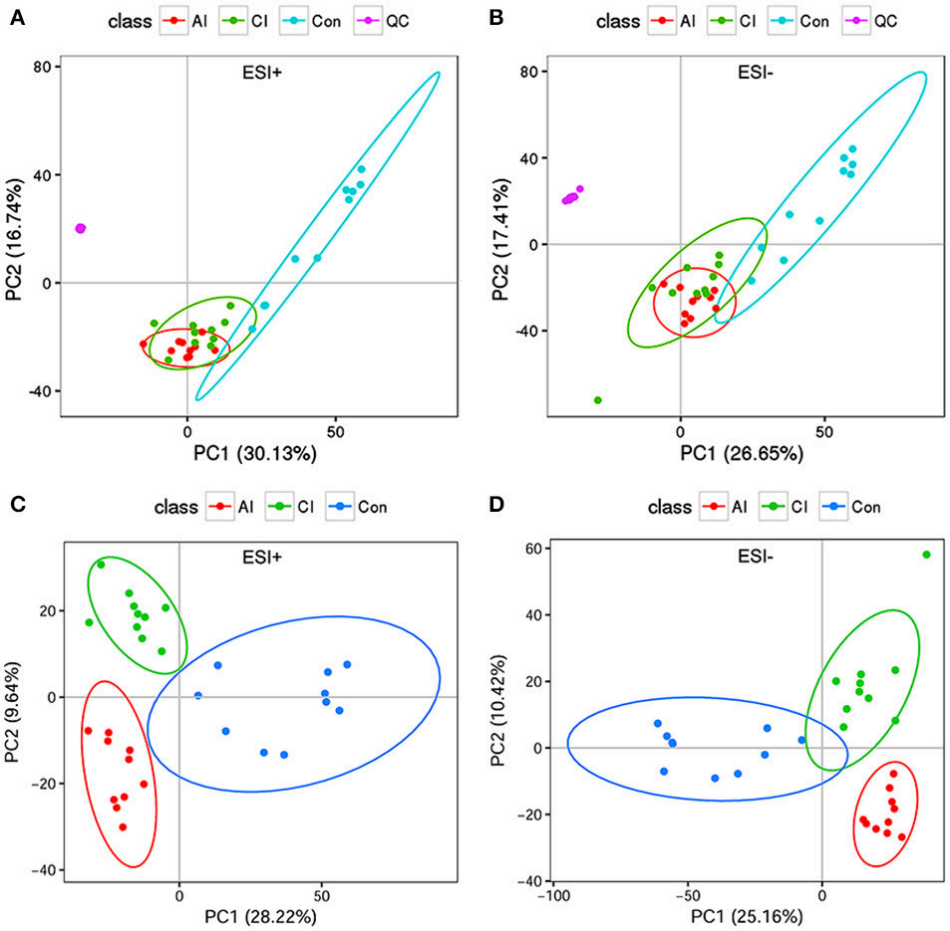
Multivariate statistical analysis of the data. **(A)** PCA scores plot of mice livers, including acutely infected (AI), chronically infected (CI) and uninfected control (Con) compared to quality control (QC) samples in the positive ion mode (ESI+). **(B)** PCA scores plot of mice livers in the negative ion mode (ESI–). Clear separation was detected among the different mice groups and in relation to QC samples. **(C)** Two dimensional PLS-DA score plots of the acutely infected (AI), chronically infected (CI) and uninfected control (Con) in the positive ion mode (ESI+). **(D)** Two dimensional PLS-DA score plots of AI, CI and Con in the negative ion mode (ESI–). Each dot represents one liver sample, projected onto first (horizontal axis) and second (vertical axis) PLS-DA variables. Mice groups are shown in different colors. The black ellipse indicates the 95% confidence interval.

To investigate the hepatic metabolic profiles during infection progression, liver extracts of *T. gondii*-infected mice and uninfected mice were analyzed with an UPLC-MS/MS system. Analysis of the total ion chromatograms (TIC) and PCA score plots of QC samples demonstrated the high stability, consistency and reproducibility of chromatographic separation as shown in Figure S4A (ESI+) and Figure S4B (ESI–). The PCA scores plot showed that the uninfected mice cluster distinctly to the right; whereas the infected mice (acutely infected and chronically infected) cluster slightly to the left without overlapping with the uninfected mice and QC samples cluster together as shown in Figure 2A (ESI+) and Figure 2B (ESI–). We also performed PLS-DA analysis to compare the three different mouse groups, including the uninfected and infected (acutely infected and chronically infected) mice, as shown in Figure 2C (ESI+) and Figure 2D (ESI–). The PLS-DA analysis showed a high degree of segregation between the various mouse groups in the positive and negative electrospray ionization modes, further supporting the reproducibility of the data.

### Differential metabolites during different infection phases

**Figure 3:**
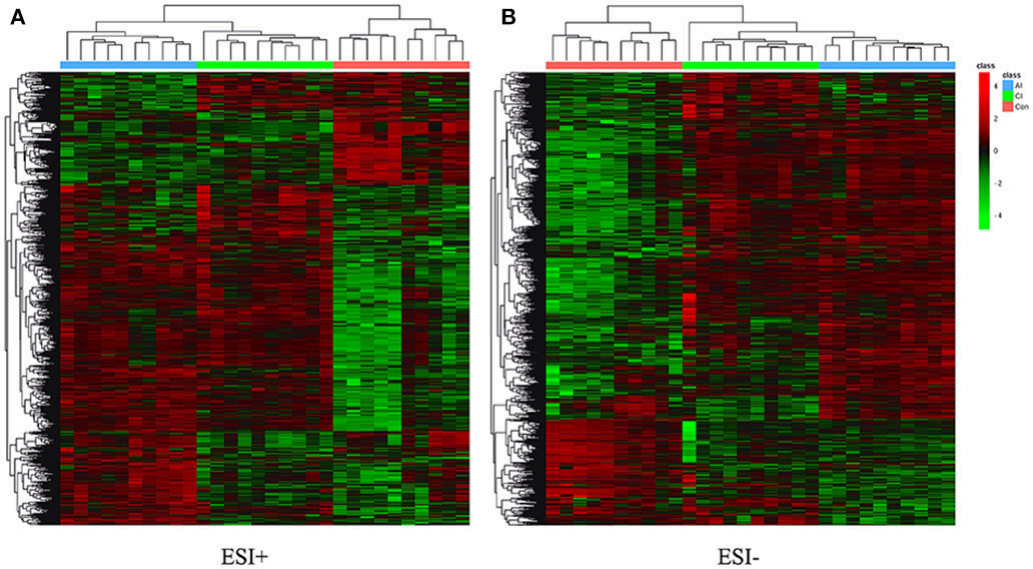
**(A)** Heatmaps of the differential metabolites in acutely infected mice (AI) and chronically infected mice (CI) vs. control mice (Con) in the positive ion mode (ESI+). **(B)** Heatmaps of the differential metabolites in acutely infected mice (AI) and chronically infected mice (CI) vs. control mice (Con) in the negative ion mode (ESI–). Red and green colors indicate values above and below the mean, respectively. Black indicates values close to the mean.

**Figure 4:**
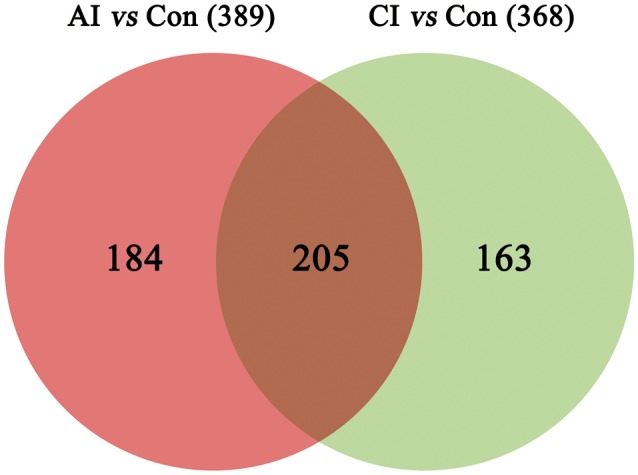
A Venn diagram showing the common and unique metabolites between the acutely and chronically infected mice vs. control. In total, 389 metabolites were found during acute infection vs. control (red); and 368 metabolites were identified in chronically infected mice vs. control (green), 205 of which were shared between the two groups.

Differential metabolites were identified on the basis of VIP ≥ 1 in the PLS-DA model and having a *q-*value < 0.05 or a fold-change ≥ 1.2 with high statistical significance (as shown in Table S1); including differential ion number, and the identified up-regulated and down-regulated ions between different experimental groups. In Table S1, we show that liver metabolic profile in acutely infected mice had the largest fold change, when compared with controls. As the infection progressed, the number of differential metabolites was relatively decreased. Meanwhile, the concentration of differential metabolites from acutely infected mice changed more significantly than those from chronically infected mice. These differential concentrations of metabolites from distinct groups were used for cluster analysis and generation of heat-maps, as shown in Figure 3A (ESI+) and Figure 3B (ESI–). The heat-maps showed distinct differences between the infected and the control mice, as well as between acutely infected and chronically infected mice. We identified 389 significant metabolites from acutely infected group vs. control (Table S2) and 368 from the chronically infected group vs. control (Table S3). A Venn diagram (Figure 4) shows the correlation between metabolites according to the infection phases, with 205 differential metabolites overlapping between the two infection phases. KEGG analysis identified 51 differential metabolites from both infection phases linked to metabolic pathways (Table 1). Further, we randomly identified several differential metabolites from Table 1, including anandamide, cholesterol, choli acid, lithocholic acid, L-Thyroxine, oleic acid and sphinganine (Figures S5–S11), using standard reference substances obtained from Sigma-Aldrich (St. Louis, USA).

**Table 1:** List of the differential metabolites involved in the perturbed metabolic pathways during acute and chronic phases of *Toxoplasma gondii* infection.

**Metabolite**	**Acute infection vs. control**			**Chronic infection vs. control**			**Metabolic pathway**
	**FC**	***q*-value**	**VIP**	**FC**	***q*-value**	**VIP**	
Cholesterol	2.83	1.85e-03	1.37	2.39	5.49e-03	1.27	Steroid biosynthesis; Primary bile acid biosynthesis; Steroid hormone biosynthesis; Hedgehog signaling pathway; Ovarian steroidogenesis; Aldosterone synthesis and secretion; Fat digestion and absorption; Bile secretion; Vitamin digestion and absorption; Pathways in cancer; Basal cell carcinoma
Ubiquinone-8	3.28	9.41e-03	1.17	2.47	9.85e-03	1.14	Ubiquinone and other terpenoid-quinone biosynthesis
2,3-Bis-O-(geranylgeranyl)-sn-glycero-1-phospho-L -serine	2.76	2.07e-02	1.25	2.23	1.07e-02	1.74	Glycerophospholipid metabolism
Taurocholic acid	0.12	3.70e-02	1.19	2.24	4.25e-02	1.30	Primary bile acid biosynthesis; Taurine and hypotaurine metabolism; Bile secretion
Dodecanoic acid	3.02	1.91e-02	1.53	3.63	7.78e-03	1.87	Fatty acid biosynthesis
Tetradecanoic acid	1.73	2.04e-03	1.16	1.70	3.62e-03	1.20	Fatty acid biosynthesis
Angiotensin III	0.28	1.56e-03	1.13	0.18	2.66e-04	1.62	Neuroactive ligand-receptor interaction; Renin-angiotensin system
L-Thyroxine	0.23	4.73e-03	1.19	0.12	5.00e-04	1.71	Tyrosine metabolism; Neuroactive ligand-receptor interaction; Thyroid hormone synthesis; Thyroid hormone signaling pathway; Bile secretion; Autoimmune thyroid disease
3-Methoxy-4-hydroxyphenylacetaldehyde	0.51	2.83e-02	1.08	0.50	2.58e-02	1.19	Tyrosine metabolism
Cholic acid	0.22	6.32e-03	1.13	0.25	1.45e-02	1.60	Primary bile acid biosynthesis; Bile secretion;
Sphinganine	2.11	1.78e-03	1.48	2.86	3.67e-02	1.73	Sphingolipid metabolism; Sphingolipid signaling pathway
Cortolone	2.72	7.21e-03	1.22	7.21	8.16e-03	2.12	Steroid hormone biosynthesis
Oleic acid	5.76	3.03e-05	2.35	2.73	1.54e-02	1.33	Fatty acid biosynthesis; Biosynthesis of unsaturated fatty acids
Sphingosine	8.13	3.14e-05	2.64	1.68	1.32e-03	1.11	Sphingolipid metabolism; Sphingolipid signaling pathway
Erucic acid	2.33	5.74e-04	1.51	1.83	4.63e-03	1.21	Biosynthesis of unsaturated fatty acids
11,12-DHET	0.06	1.82e-04	2.86	0.25	2.84e-02	1.42	Arachidonic acid metabolism; Serotonergic synapse
Docosanoic acid	3.09	6.53e-04	1.74	1.92	8.90e-03	1.29	Biosynthesis of unsaturated fatty acids
3alpha,7alpha,12alpha- Trihydroxy-5beta-cholestan-26-al	9.13	1.02e-03	1.67	5.85	1.08e-02	2.57	Primary bile acid biosynthesis
Phytosphingosine	2.06	1.25e-03	1.11	2.10	8.16e-03	1.11	Sphingolipid metabolism
Anandamide	2.24	3.16e-04	1.43	1.92	1.20e-03	1.21	Neuroactive ligand-receptor interaction; Retrograde endocannabinoid signaling; Inflammatory mediator regulation of TRP channels
Geranyl-hydroxybenzoate	2.47	5.62e-06	1.43	2.36	2.59e-05	1.53	Ubiquinone and other terpenoid-quinone biosynthesis
Palmitoleic acid	0.48	1.51e-02	1.10	7.32	1.18e-04	2.36	Fatty acid biosynthesis
Fexofenadine	2.74	5.30e-05	1.90	2.74	5.30e-05	1.90	Bile secretion
Testosterone glucuronide	1.57	3.33e-02	1.38	1.84	1.55e-02	1.69	Steroid hormone biosynthesis
2-Arachidonoylglycerol	4.08	7.26e-06	1.65	3.78	4.05e-02	1.83	Neuroactive ligand-receptor interaction; Retrograde endocannabinoid signaling
Oleoylethanolamide	0.51	5.64e-03	1.22	13.1	2.80e-03	3.74	cAMP signaling pathway
5beta-Cyprinolsulfate	6.20	4.77e-06	2.70	4.20	5.30e-05	2.19	Primary bile acid biosynthesis
2-Octaprenyl-6 -methoxyphenol	8.15	2.45e-06	1.92	5.81	3.42e-04	1.61	Ubiquinone and other terpenoid-quinone biosynthesis
Glutathionylspermidine	1.77	8.84e-03	1.21	1.58	3.39e-02	1.01	Glutathione metabolism
Vitamin D3	2.53	2.20e-03	1.26	2.59	8.83e-03	1.33	Steroid biosynthesis; Vitamin digestion and absorption; Rheumatoid arthritis
7alpha-Hydroxycholest-4 -en-3-one	2.03	6.38e-04	1.13	3.35	5.75e-03	2.13	Primary bile acid biosynthesis
Lithocholic acid	2.06	4.08e-02	1.40	0.67	1.88e-04	1.01	Bile secretion
20-Hydroxycholesterol	2.31	2.52e-03	1.21	5.90	5.44e-05	2.42	Steroid hormone biosynthesis
7-Dehydrodesmosterol	2.79	4.07e-05	1.68	2.00	2.49e-03	1.21	Steroid biosynthesis
7alpha-Hydroxycholesterol	4.72	3.78e-03	1.90	2.06	8.52e-03	1.26	Primary bile acid biosynthesis
Nervonic acid	11.1	6.37e-05	2.98	6.25	5.38e-04	2.38	Biosynthesis of unsaturated fatty acids
Bradykinin	1.94	1.95e-02	1.62	1.94	2.95e-02	1.59	cGMP-PKG signaling pathway; Sphingolipid signaling pathway; Neuroactive ligand-receptor interaction; Complement and coagulation cascades; Inflammatory mediator regulation of TRP channels; Regulation of actin cytoskeleton; Chagas disease (American trypanosomiasis); African trypanosomiasis; Pathways in cancer
20-COOH-LTB4	0.37	1.49e-02	1.60	1.90	1.78e-02	1.01	Arachidonic acid metabolism
3alpha,11beta,21-Trihydroxy-20-oxo-5beta-pregnan-18-al	0.33	3.39e-04	1.70	2.65	9.53e-03	1.36	Steroid hormone biosynthesis
Leukotriene C4	3.14	1.46e-02	2.05	3.03	2.19e-02	2.09	Arachidonic acid metabolism; Neuroactive ligand-receptor interaction; Fc epsilon RI signaling pathway; Serotonergic synapse; Bile secretion; Asthma
Delta-Tocotrienol	4.71	2.65e-04	1.64	5.17	4.88e-03	1.58	Ubiquinone and other terpenoid-quinone biosynthesis
Trypanothione disulfide	2.16	8.94e-03	1.15	2.66	3.34e-03	1.56	Glutathione metabolism
Taurolithocholate sulfate	4.44	1.43e-04	2.17	5.28	1.52e-04	2.49	Bile secretion
Icosadienoic acid	6.03	1.86e-04	1.80	4.26	4.24e-03	1.37	Biosynthesis of unsaturated fatty acids
5”-Phosphoribostamycin	0.18	1.00e-05	2.24	0.32	6.07e-04	1.54	Butirosin and neomycin biosynthesis
Trypanothione	9.45	1.81e-03	3.28	6.21	5.32e-03	2.95	Glutathione metabolism
Docosapentaenoic acid	2.70	1.87e-05	1.30	2.14	1.22e-03	1.02	Biosynthesis of unsaturated fatty acids
Pregnanolone	2.78	5.62e-03	1.16	2.27	1.77e-03	1.15	Steroid hormone biosynthesis
Phospho-anandamide	2.20	5.41e-05	1.12	2.29	1.09e-04	1.28	Retrograde endocannabinoid signaling
13,16-Docosadienoic acid	9.59	1.25e-04	2.58	4.40	1.74e-03	1.83	Biosynthesis of unsaturated fatty acids
Calcitetrol	18.3	1.75e-03	3.62	4.98	3.22e-02	2.43	Steroid biosynthesis

*FC, fold change; q-value, adjusted p value calculated by two-tailed Wilcoxon rank-sum tests after false discovery rate correction; VIP, variable importance in projection*.

### Perturbed metabolic pathways during different infection phases

**Figure 5:**
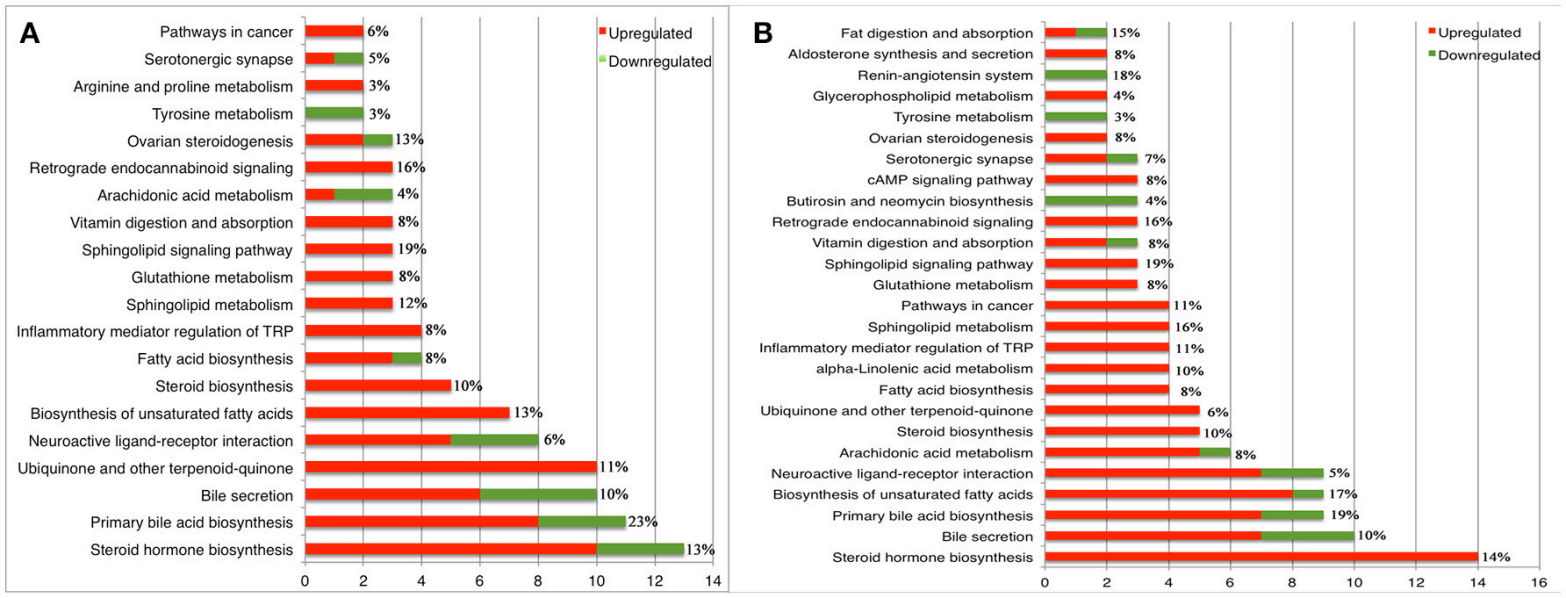
Statistics and comparison of metabolic pathways during *T. gondii* infection phases. **(A)** Metabolic pathways of enriched metabolite ≥ 2 of acutely infected group vs. control. **(B)** Metabolic pathways of enriched metabolite ≥ 2 of chronically infected vs. control. Red and green metabolites indicate higher and lower concentrations, respectively. The x-axis denotes the number of differentially abundant metabolites. The y-axis indicates the name of metabolic pathways. The percentage represents coverage rate of the pathway, which is the differential metabolites/total metabolites in the pathway.

**Figure 6:**
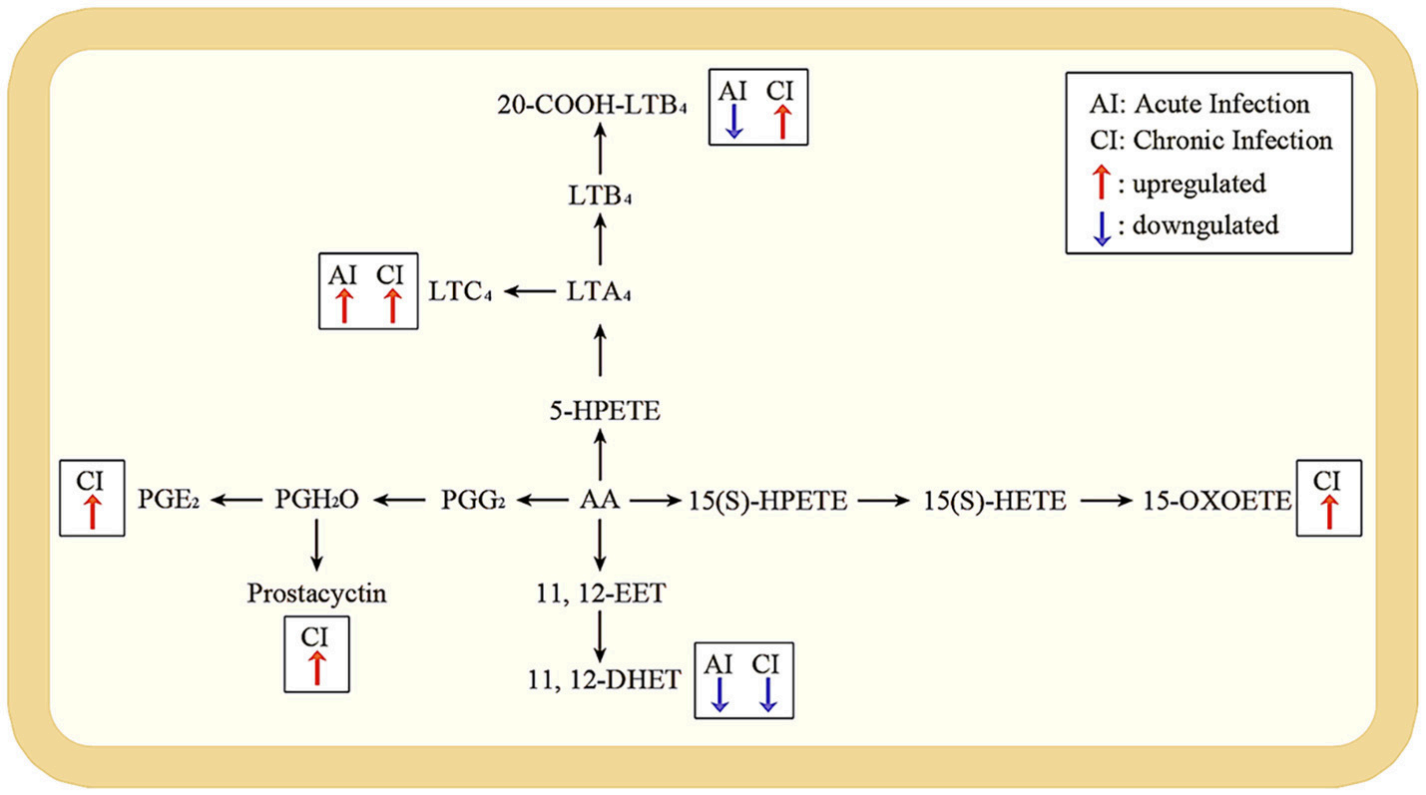
Pathway analysis of arachidonic acid metabolism during acute and chronic infection. A schematic illustration of arachidonic acid metabolism pathway; the most significant differentially expressed metabolites during acute and chronic infection phases. Red and blue arrows represent upregulated and downregulated metabolites. The metabolites without arrows are not changed. In the acute infection, Leukotriene C4 (LTC4) was upregulated whereas 20-COOH-LB4 and 11,12-DHET were downregulated; but in the chronic infection, LTC4, 20-COOH-LB4, Prostaglandin E2 (PGE2), Prostacyctin and 15-OxoETE were upregulated with only 11,12-DHET being downregulated.

According to the KEGG database annotations, there were 85 differential metabolites identified from the acutely infected group vs. control group and 92 differential metabolites from chronically infected vs. control groups, involved in metabolic pathways. The most significantly perturbed metabolic pathways and the number of upregulated and downregulated differentially abundant metabolites in each perturbed metabolic pathway, are shown (Figure 5). In the acutely infected phase: the six most perturbed metabolic pathways were involved in steroid hormone biosynthesis, primary bile acid biosynthesis, bile secretion, neuroactive ligand-receptor interaction, ubiquinone and other terpenoid-quinone biosynthesis, and the biosynthesis of unsaturated fatty acids (Figure 5A). In the chronically infected phase: steroid hormone biosynthesis, primary bile acid biosynthesis, bile secretion, neuroactive ligand-receptor interaction, biosynthesis of unsaturated fatty acids, and arachidonic acid (AA) metabolism were the six most perturbed metabolic pathways (Figure 5B). More metabolites were perturbed in AA metabolism in the chronically infected phase, when compared to the acutely infected phase. In the acutely infected phase, Leukotriene C4 was upregulated, whereas 20-COOH-LB4 and 11,12-DHET involved in AA metabolic pathway were downregulated. In the chronically infected phase, Leukotriene C4, 20-COOH-LB4, Prostaglandin E2, Prostacyctin and 15-OxoETE involved in AA metabolism were upregulated and only 11,12-DHET was downregulated. These results indicate that AA metabolism was downregulated during the acutely infected phase, but upregulated in chronic infection. A schematic illustration of the AA metabolic pathway is provided to show specific metabolites dysregulated during each infection phase (Figure 6). Multiple differentially abundant metabolites involved in amino acid metabolism were also observed (Table 2), indicating that amino acid metabolism is significantly altered during *T. gondii* infection. Multiple amino acid metabolic pathways, such as tyrosine metabolism, arginine and proline metabolism, tryptophan metabolism, histidine metabolism, glycine, serine and threonine metabolism, cysteine and methionine metabolism, taurine and hypotaurine metabolism and glutathione metabolism, were altered in the infection groups.

**Table 2:** List of the differentially expressed metabolites involved in amino acid metabolism during the acute and chronic phases of *Toxoplasma gondii* infection.

**Metabolites**	**FC**	***q*-value**	**VIP**	**CV**	**Metabolic pathways**
**ACUTELY INFECTED VS. CONTROL**					
L-Thyroxine	0.23	4.73e-03	1.19	↓	Tyrosine metabolism
3-Methoxy-4-hydroxyphenylacetaldehyde	0.51	2.83e-02	1.08	↓	
gamma-Glutamyl-gamma-aminobutyraldehyde	2.59	4.45e-03	1.80	↑	Arginine and proline metabolism
Nopaline	1.84	6.92e-03	1.21	↑	
Indole-3-ethanol	2.17	5.45e-03	1.38	↑	Tryptophan metabolism
Taurocholate	0.12	3.70e-02	1.20	↓	Taurine and hypotaurine metabolism
Glutathionylspermidine	1.77	8.84e-03	1.21	↑	Glutathione metabolism
Trypanothione disulfide	2.16	8.94e-03	1.15	↑	
Trypanothione	9.45	1.81e-03	3.28	↑	
**CHRONICALLY INFECTED VS. CONTROL**					
L-Thyroxine	0.12	5.00e-04	1.71	↓	Tyrosine metabolism
3-Methoxy-4-hydroxyphenylacetaldehyde	0.50	2.58e-02	1.19	↓	
L-Cystathionine	0.51	2.08e-02	1.21	↓	Glycine, serine and threonine metabolism; Cysteine and methionine metabolism
N-Formyl-L-aspartate	0.65	3.73e-04	1.03	↓	Histidine metabolism
Glutathionylspermidine	1.58	3.39e-02	1.01	↑	Glutathione metabolism
Trypanothione disulfide	2.66	3.34e-03	1.56	↑	
Trypanothione	6.21	5.32e-03	2.95	↑	

*FC, Fold change; q-value, adjusted p value calculated by the two-tailed Wilcoxon rank-sum tests after false discovery rate correction; VIP, variable importance in projection; CV, Content variance. A single arrow (↑/↓) indicates increased or decreased values compared to the control group*.

## Discussion

A non-targeted metabolomics-based approach is presented that enabled the study of liver metabolites and metabolic pathways during *T. gondii* infection with the aim of defining infection-specific and stage-specific metabolic signatures. This approach revealed 389 and 368 significantly altered metabolites in acutely infected mice and chronically infected mice, respectively, vs. control. The decreased number of altered metabolites during late infection indicates the restoration of the metabolic state. 205 differential metabolites were common to both acutely and chronically infected mice, of these 51 differential metabolites were involved in metabolic pathways (Table 1).

Previous LC-MS metabolomics analysis of mouse brain detected 60 differentially abundant metabolites at different stages of *T. gondii* infection (Zhou et al., 2015). Liquid chromatography-quadrupole time-of-flight mass spectrometry (LC-Q-TOF-MS)-based metabolomics analysis identified 79 and 74 differentially abundant metabolites in ESI+ mode and ESI– mode, respectively, in sera of *T. gondii*-infected mice compared to controls (Zhou et al., 2016). LC-MS/MS-based metabolomics analysis of *T. gondii*-infected mouse spleen revealed 132 differentially abundant metabolites (23 metabolites from acutely infected mice and 109 metabolites from chronically infected mice) compared with control (Chen et al., 2017). This discord between metabolic phenotyping of *T. gondii* infected tissues can be attributed to heterogeneity in host tissue types or to variation in the experimental conditions and the analytical protocols used in these studies.

The liver response to infection should be strong enough to control the parasite and in the meantime restrained in order to minimize immune-inflammatory pathology. Achieving this balance between protection against infection and immunopathology-induced tissue damage is essential in order to limit liver injury during *T. gondii* infection. We identified a number of altered metabolites and metabolic pathways involved in the inflammatory response to *T. gondii* infection. Taurine, the most abundant free amino acid in the liver and other tissues, has antioxidant and anti-inflammatory activities (Rosa et al., 2014). Reduced level of taurocholic acid that is involved in bile acid biosynthesis, taurine and hypotaurine metabolism, was detected during acute infection, probably to facilitate the parasite establishment. This taurine-related reduction in the anti-inflammatory status seemed to be buffered by increased levels of glutathionylspermidine, trypanothione disulfide and trypanothione metabolites involved in glutathione (GSH) metabolism during acute and chronic infections (Silva et al., 2002; Marino and Boothroyd, 2017). GSH is an important antioxidant that plays an important role in the defense against reactive oxygen species produced during oxidative metabolism, and contributes to DNA repair and protein synthesis (Dringen, 2000; Canugovi et al., 2013). It is important to note that trypanothione and trypanothione reductase are unique to the trypanosomatid protozoa (Vincent et al., 2016), as the main defense mecahnism against oxidative damage (Fiorillo et al., 2012). Because *T. gondii* lacks the genes for trypanothione biosynthesis, it is possible that trypanothione metabolites were misidentified in the present study. It is also possible that *T. gondii* synthesizes trypanosome-specific trypanothione metabolite using an as yet undiscovered route, but this assumption remains to be confirmed.

Infection also induced significant changes in levels of metabolites involved in amino acid metabolism (Table 2). Tryptophan, tyrosine and arginine are essential amino acids required by the parasite to sustain its own growth (Silva et al., 2002; Marino and Boothroyd, 2017). In both acutely and chronically infected mice, L-thyroxine and 3-Methoxy-4-hydroxyphenylacetaldehyde, involved in tyrosine metabolism, were downregulated. Uniquely in acutely infected mice, indole-3-ethanol involved in tryptophan metabolism, and gamma-glutamyl-gamma-aminobutyraldehyde and nopaline involved in arginine and proline metabolism, were upregulated. This finding is consistent with our earlier proteomic analysis of mouse liver infected with *T. gondii*, where indoleamine 2,3-dioxygenase (Ido2) was downregulated in the infected livers, probably to support the parasite growth by countering the reduction in tryptophan (He et al., 2016b). Dysregulation in the levels of these amino acids is to be anticipated because this parasite is auxotrophic for tryptophan and arginine, among other amino acids.

We previously showed, based on preotomics analysis, that *T. gondii* interferes with the metabolism of fatty acids, lipids and energy in the liver via the modulation of host peroxisome proliferator-activated receptor (PPAR) signaling pathway (He et al., 2016b). We also observed notable changes in the hepatic levels of lipids in infected mice. As shown in Figure 5 and Table 1, in acutely and chronically infected mice, the major perturbed metabolic pathways included steroid hormone biosynthesis, primary bile acid biosynthesis, steroid biosynthesis, biosynthesis of unsaturated fatty acids, fatty acid biosynthesis, and arachidonic acid metabolism. Downregulation of genes involved in bile synthesis, and the metabolism of sterol, fatty acids, and lipids have also been reported (He et al., 2016a). Steroid hormones contribute to several physiological processes; relevant to *T. gondii* infection are the immunoregulatory activities of steroids, which can influence the host immune response to infection (Tait et al., 2008). Fatty acids are essential for the synthesis of membranes of the newly produced parasites (Ramakrishnan et al., 2012) and lipids have been implicated in the pathogenesis of *T. gondii* infection (Milovanović et al., 2017). Upregulation of metabolites involved in sphingolipid metabolism was also observed during both phases of infection. Sphingolipids play critical roles in mediating cell-cell interaction, modulating the behavior of cellular proteins and receptors, and participate in signal transduction (Merrill, 2002).

Arachidonic acid (AA) metabolism was the most significant differentially abundant metabolic pathway between acutely and chronically infected mice. AA has been shown to promote *T. gondii* invasion (Li et al., 2008). The metabolism of AA was downregulated during acute infection and upregulated during chronic infection (Figure 6). In response to an inflammatory stimulus, AA, the main polyunsaturated fatty acid present in the phospholipid of cell membranes, is released and metabolized to a series of eicosanoids, including the inflammatory leukotrienes and prostaglandins (Greene et al., 2011). AA and its inflammatory metabolites mediate the regulation of key cellular processes, such as cell survival, angiogenesis, chemotaxis, mitogenesis, apoptosis and migration (Rizzo et al., 1999; Levick et al., 2007). Alpha-linolenic acid (ALA), an essential omega-3 fatty acid that is commonly found in seeds, nuts, and vegetable oils, was perturbed during chronic stage of infection. However, because ALA is an essential fatty acid (i.e., cannot be synthesized by the mice), its increased level must have been derived from the mouse diet.

Our results showed a stronger impact of the parasite infection on the liver metabolic status during acute compared to chronic infection. However, one possible reason for the metabolic alterations observed during chronic infection may be partly related to considering 30 dpi as the chronic phase of *T. gondii* infection, which is a relatively early time point to mark the latency of *T. gondii* infection. In fact, a previous study showed low parasite burden and absence of parasite DNA in the liver of Swiss albino mice infected with *T. gondii* type II Me49 strain at 28 and 42 dpi, respectively (Djurković-Djaković et al., 2012).

## Conclusion

By taking a global approach to understanding the metabolomics of mouse liver in the context of *T. gondii* infection, we have identified a number of previously known and novel metabolites that play biological roles relevant to host-parasite interaction. Our data indicate that mouse liver harbors intrinsic mechanisms to restrict *T. gondii* replication. For example, through the upregulation of GSH metabolism, liver can buffer oxidative stress associated with infection. On the other hand, the parasite has evolved mechanisms to exploit various lipids and amino acids, to support its own proliferation. Our metabolic profiling also showed many altered metabolites and metabolic pathways (e.g., steroid hormone biosynthesis, primary bile acid biosynthesis, bile secretion, and biosynthesis of unsaturated fatty acids) in the liver of *T. gondii*-infected mice during acute and chronic infections when compared with healthy uninfected mice. These findings provide new insight into metabolic changes that occur in the liver in acute and chronic murine toxoplasmosis.

## Data availability statement

The datasets supporting the findings of this article are included within the article. The metabolomics data are available in the MetaboLights database (MTBLS586) (http://www.ebi.ac.uk/metabolights/).

## Author contributions

X-QZ and C-XZ conceived and designed the experiments. X-QC and C-XZ performed the experiments. X-QC analyzed the data and wrote the paper. R-SH, G-XH, and S-LG helped in the analyzing of the data. X-QC, HE and X-QZ critically revised the manuscript. All authors read and approved the final version of the manuscript.

### Conflict of interest statement

The authors declare that the research was conducted in the absence of any commercial or financial relationships that could be construed as a potential conflict of interest.

## Supplementary material

Figure S1 The original image of Figure 1A.

Figure S2 The original image of Figure 2B.

Figure S3 The original image of Figure 3C.

Figure S4 (A) Total ion current (TIC) chromatograms of liver samples in the positive ion mode (ESI+). **(B)** Total ion current (TIC) chromatograms of liver samples in the negative ion mode (ESI–).

Figure S5 Secondary spectrogram information of anandamide. **(A)** Standard secondary spectrogram of anandamide in the positive ion mode (ESI+). **(B)** Secondary spectrogram of experimental samples at similar retention time in the positive ion mode (ESI+).

Figure S6 Secondary spectrogram information of cholesterol. **(A)** Standard secondary spectrogram of cholesterol in the positive ion mode (ESI+). **(B)** Secondary spectrogram of experimental samples at similar retention time in the positive ion mode (ESI+).

Figure S7 Secondary spectrogram information of choli acid. **(A)** Standard secondary spectrogram of choli acid in the positive ion mode (ESI+). **(B)** Secondary spectrogram of experimental samples at similar retention time in the positive ion mode (ESI+).

Figure S8 Secondary spectrogram information of lithocholic acid. **(A)** Standard secondary spectrogram of lithocholic acid in the positive ion mode (ESI+). **(B)** Secondary spectrogram of experimental samples at similar retention time in the positive ion mode (ESI+).

Figure S9 Secondary spectrogram information of L-Thyroxine. **(A)** Standard secondary spectrogram of L-Thyroxine in the positive ion mode (ESI+). **(B)** Secondary spectrogram of experimental samples at similar retention time in the positive ion mode (ESI+).

Figure S10 Secondary spectrogram information of oleic acid. **(A)** Standard secondary spectrogram of oleic acid in the positive ion mode (ESI+). **(B)** Secondary spectrogram of experimental samples at similar retention time in the positive ion mode (ESI+).

Figure S11 Secondary spectrogram information of sphinganine. **(A)** Standard secondary spectrogram of sphinganine in the positive ion mode (ESI+). **(B)** Secondary spectrogram of experimental samples at similar retention time in the positive ion mode (ESI+).

Table S1 Summary results of the differential ions.

Table S2 List of metabolites identified during acute *Toxoplasma gondii* infection.

Table S3 List of metabolites identified during chronic *Toxoplasma gondii* infection.

## References

[B1] Antunes L. C. Arena E. T. Menendez A. Han J. Ferreira R. B. Buckner M. M.. (2011). Impact of *Salmonella* infection on host hormone metabolism revealed by metabolomics. Infect. Immun. 79, 1759–1769. 10.1128/IAI.01373-10 21321075 PMC3067560

[B2] Canugovi C. Misiak M. Ferrarelli L. K. Croteau D. L. Bohr V. A. (2013). The role of DNA repair in brain related disease pathology. DNA repair (Amst) 12, 578–587. 10.1016/j.dnarep.2013.04.010 23721970 PMC3720728

[B3] Charron A. J. Sibley L. D. (2002). Host cells: mobilizable lipid resources for the intracellular parasite *Toxoplasma gondii*. J. Cell Sci. 115, 3049–3059. 12118061 10.1242/jcs.115.15.3049

[B4] Chaudhary K. Darling J. A. Fohl L. M. Sullivan W. J. Jr. Donald R. G. Pfefferkorn E. R.. (2004). Purine salvage pathways in the apicomplexan parasite *Toxoplasma gondii*. J. Biol. Chem. 279, 31221–31227. 10.1074/jbc.M404232200 15140885

[B5] Chen X. Q. Zhou C. X. Elsheikha H. M. He S. Hu G. X. Zhu X. Q. (2017). Profiling of the perturbed metabolomic state of mouse spleen during acute and chronic toxoplasmosis. Parasite Vectors 10:339. 10.1186/s13071-017-2282-6 28720125 PMC5516376

[B6] Cong W. Zhang X. X. He J. J. Li F. C. Elsheikha H. M. Zhu X. Q. (2017). Global miRNA expression profiling of domestic cat livers following acute *Toxoplasma gondii* infection. Oncotarget 8, 25599–25611. 10.18632/oncotarget.16108 28424428 PMC5421954

[B7] Coppens I. Joiner K. A. (2003). Host but not parasite cholesterol controls *Toxoplasma* cell entry by modulating organelle discharge. Mol. Biol. Cell 14, 3804–3820. 10.1091/mbc.e02-12-0830 12972565 PMC196568

[B8] Coppens I. Sinai A. P. Joiner K. A. (2000). *Toxoplasma gondii* exploits host low-density lipoprotein receptor- mediated endocytosis for cholesterol acquisition. J. Cell Biol. 149, 167–180. 10.1083/jcb.149.1.167 10747095 PMC2175092

[B9] Dai W. Pan H. Kwok O. Dubey J. P. (1994). Human indoleamine 2,3-dioxygenase inhibits *Toxoplasma gondii* growth in fibroblast cells. J. Interferon Res. 14, 313–317. 10.1089/jir.1994.14.313 7897249

[B10] Dimier I. H. Bout D. T. (1998). Interferon-gamma-activated primary enterocytes inhibit *Toxoplasma gondii* replication: a role for intracellular iron. Immunol. 94, 488–495. 10.1046/j.1365-2567.1998.00553.x 9767436 PMC1364226

[B11] Djurković-Djaković O. Djokić V. Vujanić M. Zivković T. Bobić B. Nikolić A.. (2012). Kinetics of parasite burdens in blood and tissues during murine toxoplasmosis. Exp. Parasitol. 131, 372–376. 10.1016/j.exppara.2012.05.006 22613495

[B12] Dringen R. (2000). Metabolism and functions of glutathione in brain. Prog Neurobiol. 62, 649–671. 10.1016/S0301-0082(99)00060-X 10880854

[B13] Dubey J. P. (2008). The history of *Toxoplasma gondii*–the first 100 years. J. Eukaryot. Microbiol. 55, 467–475. 10.1111/j.1550-7408.2008.00345.x 19120791

[B14] Dubey J. P. Beattie C. P. (1988). Toxoplasmosis of Animals and Man. Boca Raton, FL: CRC Press.

[B15] Dubey J. P. Frenkel J. K. (1976). Feline toxoplasmosis from acutely infected mice and the development of *Toxoplasma* cysts. J. Protozool. 23, 537–546. 10.1111/j.1550-7408.1976.tb03836.x 1003342

[B16] Dupont C. D. Christian D. A. Hunter C. A. (2012). Immune response and immunopathology during toxoplasmosis. Semin. Immunopathol. 34, 793–813. 10.1007/s00281-012-0339-3 22955326 PMC3498595

[B17] Elsheikha H. M. (2008). Congenital toxoplasmosis: priorities for further health promotion action. Public Health 122, 335–353. 10.1016/j.puhe.2007.08.009 17964621

[B18] Elsheikha H. M. Zhu X. Q. (2016). *Toxoplasma gondii* infection and schizophrenia: an inter-kingdom communication perspective. Curr. Opin. Infect. Dis. 29, 311–318. 10.1097/QCO.0000000000000265 27120002

[B19] Fiorillo A. Colotti G. Boffi A. Baiocco P. Ilari A. (2012). The crystal structures of the tryparedoxin-tryparedoxin peroxidase couple unveil the structural determinants of *Leishmania* detoxification pathway. PLoS Negl. Trop. Dis. 6:e1781. 10.1371/journal.pntd.0001781 22928053 PMC3424247

[B20] Fox B. A. Gigley J. P. Bzik D. J. (2004). *Toxoplasma gondii* lacks the enzymes required for de novo arginine biosynthesis and arginine starvation triggers cyst formation. Int. J. Parasitol. 34, 323–331. 10.1016/j.ijpara.2003.12.001 15003493

[B21] Greene E. R. Huang S. Serhan C. N. Panigrahy D. (2011). Regulation of inflammation in cancer by eicosanoids. Prostaglandins Other Lipid Mediat. 96, 27–36. 10.1016/j.prostaglandins.2011.08.004 21864702 PMC4051344

[B22] He J. J. Ma J. Elsheikha H. M. Song H. Q. Zhou D. H. Zhu X. Q. (2016b). Proteomic profiling of Mouse liver following acute *Toxoplasma gondii* infection. PLoS ONE 11:e0152022. 10.1371/journal.pone.0152022 27003162 PMC4803215

[B23] He J. J. Ma J. Elsheikha H. M. Song H. Q. Zhu X. Q. (2016a). Transcriptomic analysis of mouse liver reveals a potential hepato-enteric pathogenic mechanism in acute *Toxoplasma gondii* infection. Parasit. Vectors 9:427. 10.1186/s13071-016-1716-x 27488578 PMC4973073

[B24] Hill D. E. Chirukandoth S. Dubey J. P. Lunney J. K. Gamble H. R. (2006). Comparison of detection methods for *Toxoplasma gondii* in naturally and experimentally infected swine. Vet. Parasitol. 141, 9–17. 10.1016/j.vetpar.2006.05.008 16815636

[B25] Holmes E. Wilson I. D. Nicholson J. K. (2008). Metabolic phenotyping in health and disease. Cell 134, 714–717. 10.1016/j.cell.2008.08.026 18775301

[B26] Kafsack B. F. Llinás M. (2010). Eating at the table of another: metabolomics of host-parasite interactions. Cell Host Microbe. 7, 90–99. 10.1016/j.chom.2010.01.008 20159614 PMC2825149

[B27] Levick S. P. Loch D. C. Taylor S. M. Janicki J. S. (2007). Arachidonic acid metabolism as a potential mediator of cardiac fibrosis associated with inflammation. J. Immunol. 178, 641–646. 10.4049/jimmunol.178.2.641 17202322

[B28] Li L. Li X. Yan J. (2008). Alterations of concentrations of calcium and arachidonic acid and agglutinations of microfilaments in host cells during *Toxoplasma gondii* invasion. Vet. Parasitol. 157, 21–23. 10.1016/j.vetpar.2008.07.007 18706765

[B29] Marino N. D. Boothroyd J. C. (2017). *Toxoplasma* growth *in vitro* is dependent on exogenous tyrosine and is independent of AAH2 even in tyrosine-limiting conditions. Exp. Parasitol. 176, 52–58. 10.1016/j.exppara.2017.02.018 28257757 PMC5423395

[B30] Merrill A. H. Jr. (2002). De novo sphingolipid biosynthesis: a necessary, but dangerous, pathway. J. Biol. Chem. 277, 25843–25846. 10.1074/jbc.R200009200 12011104

[B31] Milovanović I. Busarcević M. Trbovich A. Ivović V. Uzelac A. (2017). Evidence for host genetic regulation of altered lipid metabolism in experimental toxoplasmosis supported with gene data mining results. PLoS ONE 12:e0176700. 10.1371/journal.pone.0176700 28459857 PMC5411058

[B32] Munoz M. Liesenfeld O. Heimesaat M. M. (2011). Immunology of *Toxoplasma gondii*. Immunol. Rev. 240, 269–285. 10.1111/j.1600-065X.2010.00992.x 21349099

[B33] Naemat A. Elsheikha H. M. Boitor R. A. Notingher I. (2016). Tracing amino acid exchange during host-pathogen interaction by combined stable-isotope time-resolved Raman spectral imaging. Sci. Rep. 6:20811. 10.1038/srep20811 26857158 PMC4746650

[B34] Nelson M. M. Jones A. R. Carmen J. C. Sinai A. P. Burchmore R. Wastling J. M. (2008). Modulation of the host cell proteome by the intracellular apicomplexan parasite *Toxoplasma gondii*. Infect. Immun. 76, 828–844. 10.1128/IAI.01115-07 17967855 PMC2223483

[B35] Nguyen C. T. Shetty V. Maresso A. W. (2015). Global metabolomic analysis of a mammalian host infected with *Bacillus anthracis*. Infect. Immun. 83, 4811–4825. 10.1128/IAI.00947-15 26438791 PMC4645395

[B36] Nicholson J. K. Lindon J. C. Holmes E. (1999). ‘Metabonomics’: understanding the metabolic responses of living systems to pathophysiological stimuli via multivariate statistical analysis of biological NMR spectroscopic data. Xenobiotica 29, 1181–1189. 10598751 10.1080/004982599238047

[B37] Pfefferkorn E. R. (1984). Interferon gamma blocks the growth of *Toxoplasma gondii* in human fibroblasts by inducing the host cells to degrade tryptophan. Proc. Natl. Acad. Sci. U.S.A. 81, 908–912. 10.1073/pnas.81.3.908 6422465 PMC344948

[B38] Pittman K. J. Aliota M. T. Knoll L. J. (2014). Dual transcriptional profiling of mice and *Toxoplasma gondii* during acute and chronic infection. BMC Genomics 15:806. 10.1186/1471-2164-15-806 25240600 PMC4177681

[B39] Ramakrishnan S. Docampo M. D. Macrae J. I. Pujol F. M. Brooks C. F. van Dooren G. G.. (2012). Apicoplast and endoplasmic reticulum cooperate in fatty acid biosynthesis in apicomplexan parasite *Toxoplasma gondii*. J. Biol. Chem. 287, 4957–4971. 10.1074/jbc.M111.310144 22179608 PMC3281623

[B40] Rizzo M. T. Regazzi E. Garau D. Akard L. Dugan M. Boswell H. S.. (1999). Induction of apoptosis by arachidonic acid in chronic myeloid leukemia cells. Cancer Res. 59, 5047–5053. 10519422

[B41] Rosa F. T. Freitas E. C. Deminice R. Jordão A. A. Marchini J. S. (2014). Oxidative stress and inflammation in obesity after taurine supplementation: a double-blind, placebo-controlled study. Eur. J. Nutr. 53, 823–830. 10.1007/s00394-013-0586-7 24065043

[B42] Schwartzman J. D. Pfefferkorn E. R. (1982). *Toxoplasma gondii*: purine synthesis and salvage in mutant host cells and parasites. Exp. Parasitol. 53, 77–86. 10.1016/0014-4894(82)90094-7 7198995

[B43] Seabra S. H. DaMatta R. A. de Mello F. G. de Souza W. (2004). Endogenous polyamine levels in macrophages is sufficient to support growth of *Toxoplasma gondii*. J. Parasitol. 90, 455–460. 10.1645/GE-179R 15270085

[B44] Shin J. H. Yang J. Y. Jeon B. Y. Yoon Y. J. Cho S. N. Kang Y. H. (2011). 1H NMR-based metabolomic profiling in mice infected with *Mycobacterium tuberculosis*. J. Proteome Res. 10, 2238–2247. 10.1021/pr101054m 21452902

[B45] Silva N. M. Rodrigues C. V. Santoro M. M. (2002). Expression of indoleamine 2,3-dioxygenase, tryptophan degradation, and kynurenine formation during *in vivo* infection with *Toxoplasma gondii*: induction by endogenous gamma interferon and requirement of interferon regulatory factor 1. Infect. Immun. 70, 859–868. 10.1128/IAI.70.2.859-868.2002 11796621 PMC127654

[B46] Sullivan W. J. Jr. Jeffers V. (2012). Mechanisms of *Toxoplasma gondii* persistence and latency. FEMS Microbiol. Rev. 36, 717–733. 10.1111/j.1574-6976.2011.00305.x 22091606 PMC3319474

[B47] Tait A. S. Butts C. L. Sternberg E. M. (2008). The role of glucocorticoids and progestins in inflammatory, autoimmune, and infectious disease. J. Leukoc. Biol. 84, 924–931. 10.1189/jlb.0208104 18664528 PMC2538604

[B48] Vincent I. M. Daly R. Courtioux B. Cattanach A. M. Biéler S. Ndung'u J. M.. (2016). Metabolomics identifies multiple candidate biomarkers to diagnose and stage human african trypanosomiasis. PLoS Negl. Trop. Dis. 10:e0005140. 10.1371/journal.pntd.0005140 27941966 PMC5152828

[B49] Vischer T. L. Bernheim C. Engelbrecht E. (1967). Two cases of hepatitis due to *Toxoplasma gondii*. Lancet 290, 919–921. 10.1016/S0140-6736(67)90235-8 4167857

[B50] Weitberg A. B. Alper J. C. Diamond I. Fligiel Z. (1979). Acute granulomatous hepatitis in the course of acquired toxoplasmosis. N. Engl. J. Med. 300, 1093–1096. 10.1056/NEJM197905103001907 431613

[B51] Wen B. Mei Z. Zeng C. Liu S. (2017). metaX: a flexible and comprehensive software for processing metabolomics data. BMC Bioinformatics 18:183. 10.1186/s12859-017-1579-y 28327092 PMC5361702

[B52] Wendum D. Carbonell N. Svrcek M. Chazouilléres O. Fléjou J. (2002). Fatal disseminated toxoplasmosis in a *toxoplasma* seropositive liver transplant recipient. J. Clin. Pathol. 55:637. 10.1136/jcp.55.8.637 12147667 PMC1769724

[B53] Yang J. Zhao X. Liu X. Wang C. Gao P. Wang J.. (2006). High performance liquid chromatography-mass spectrometry for metabonomics: potential biomarkers for acute deterioration of liver function in chronic hepatitis B. J. Proteome Res. 5, 554–561. 10.1021/pr050364w 16512670

[B54] Zhong H. Fang C. Fan Y. Lu Y. Wen B. Ren H.. (2017). Lipidomic profiling reveals distinct differences in plasma lipid composition in healthy, prediabetic and type 2 diabetic individuals. Gigascience 6, 1–12. 10.1093/gigascience/gix036 28505362 PMC5502363

[B55] Zhou C. X. Zhou D. H. Elsheikha H. M. Liu G. X. Suo X. Zhu X. Q. (2015). Global metabolomic profiling of mice brains following experimental infection with the cyst-forming *Toxoplasma gondii*. PLoS ONE 10:e0139635. 10.1371/journal.pone.0139635 26431205 PMC4592003

[B56] Zhou C. X. Zhou D. H. Elsheikha H. M. Zhao Y. Suo X. Zhu X. Q. (2016). Metabolomic profiling of mice serum during toxoplasmosis progression using liquid chromatography-mass spectrometry. Sci. Rep. 6:19557. 10.1038/srep19557 26785939 PMC4726199

