# Supplementary figures and images for: Hepatic Metabolomics Investigation in Acute and Chronic Murine Toxoplasmosis
Source: Front Cell Infect Microbiol. 2018 Jun 5;8:189. doi: 10.3389/fcimb.2018.00189 (PMC5996072; doi:10.3389/fcimb.2018.00189)

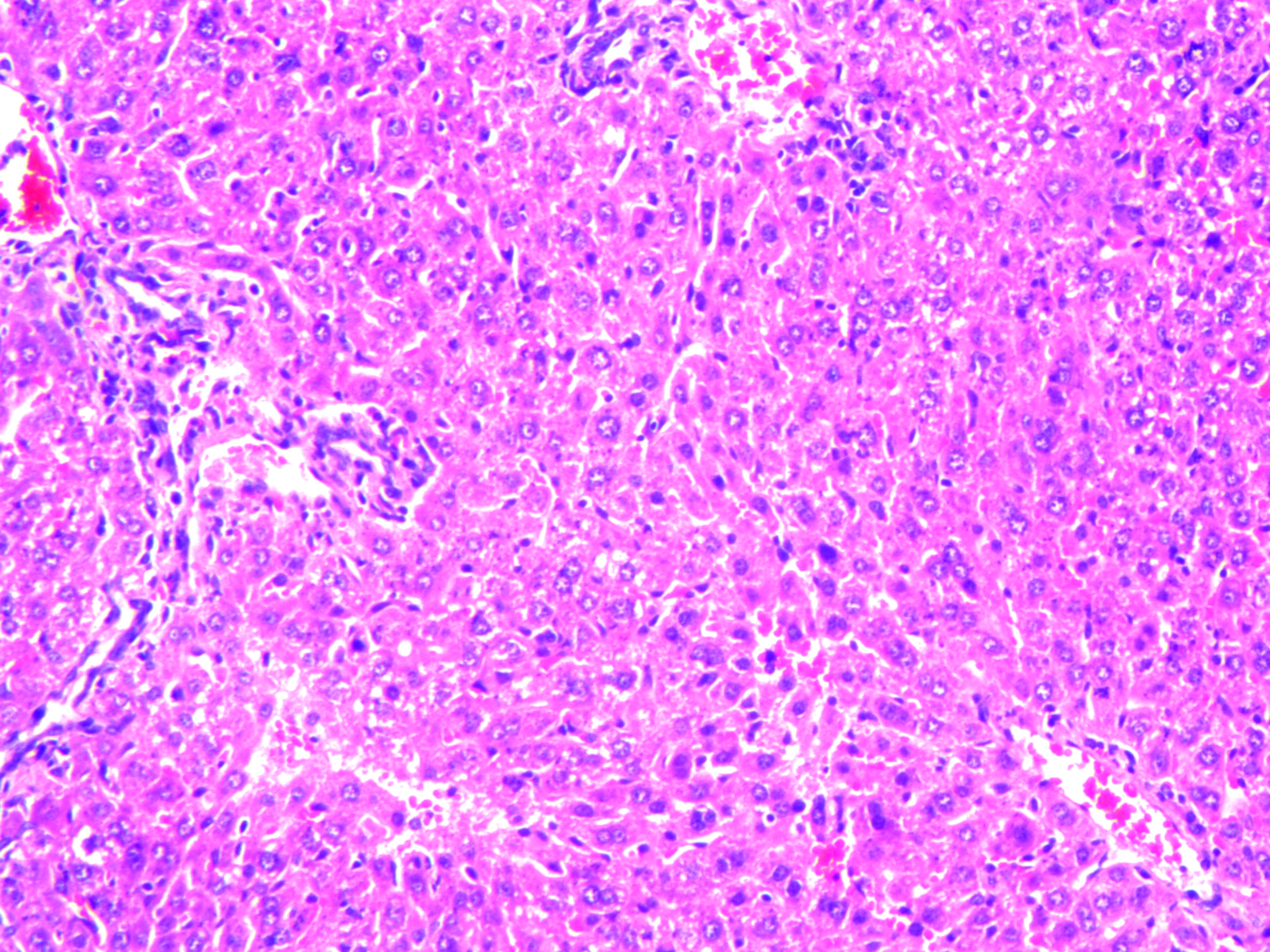

Supplement: Figure S1 — The original image of Figure 1A. [file Image_1.TIF]

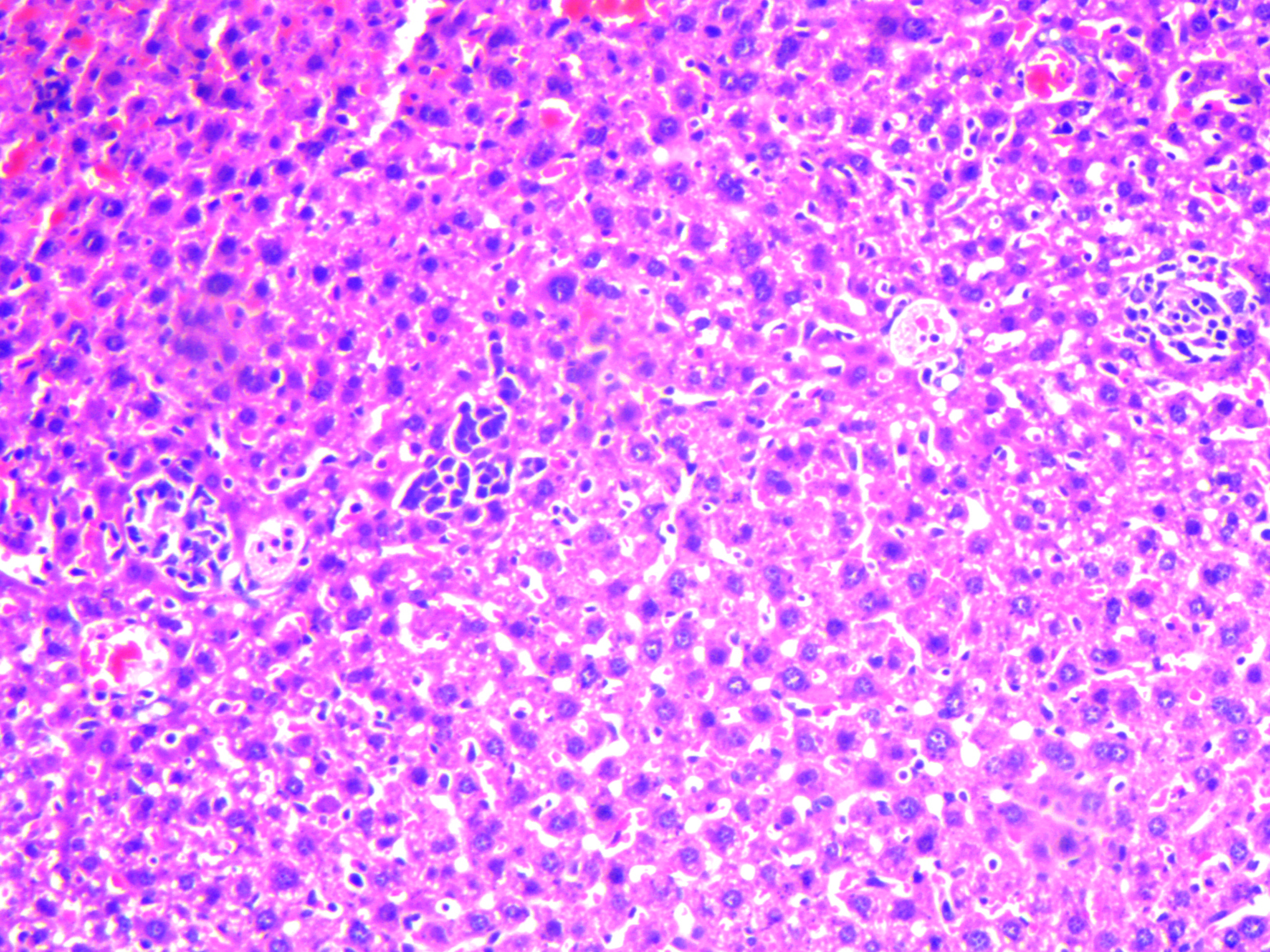

Supplement: Figure S2 — The original image of Figure 2B. [file Image_2.TIF]

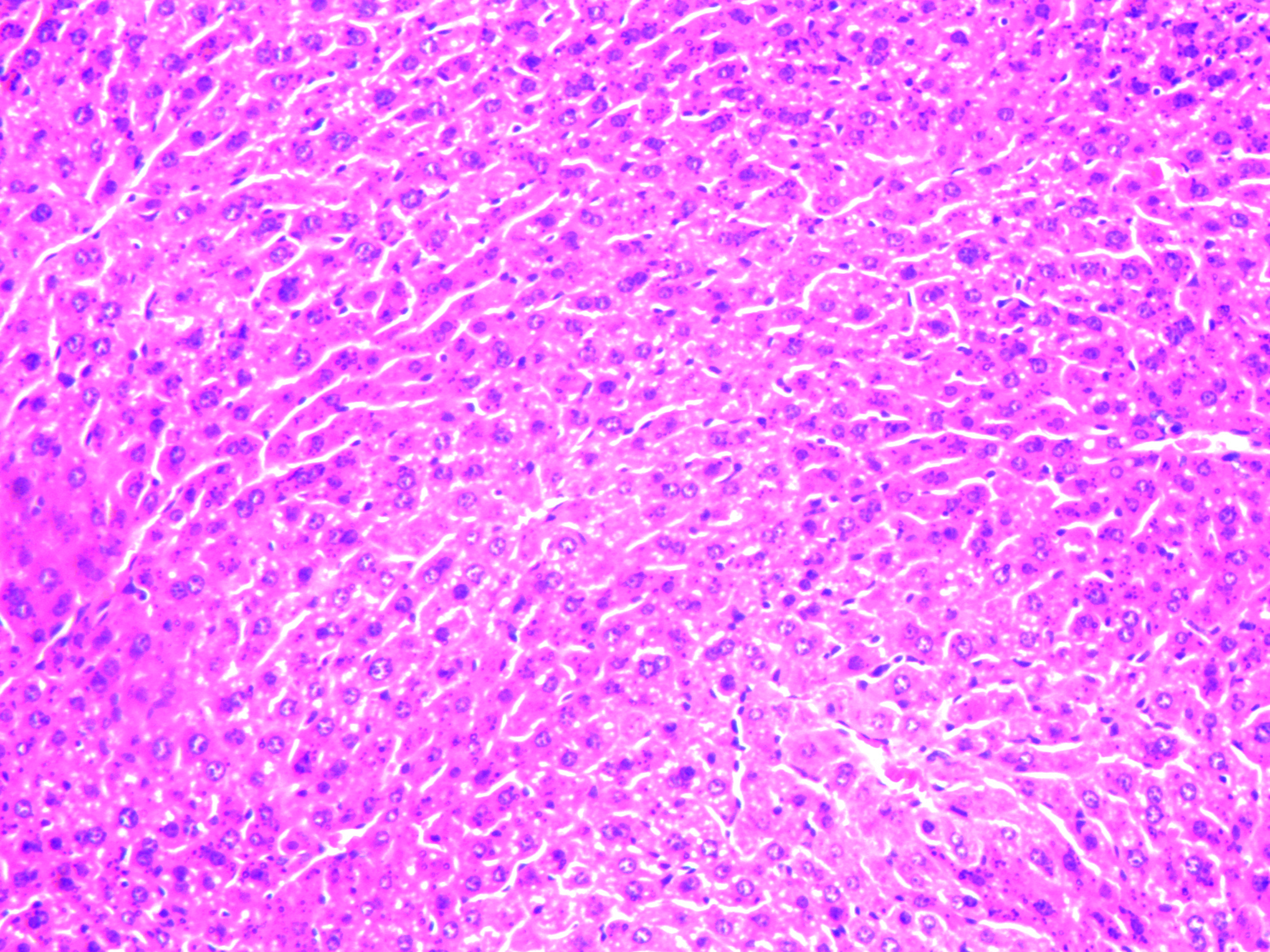

Supplement: Figure S3 — The original image of Figure 3C. [file Image_3.TIF]

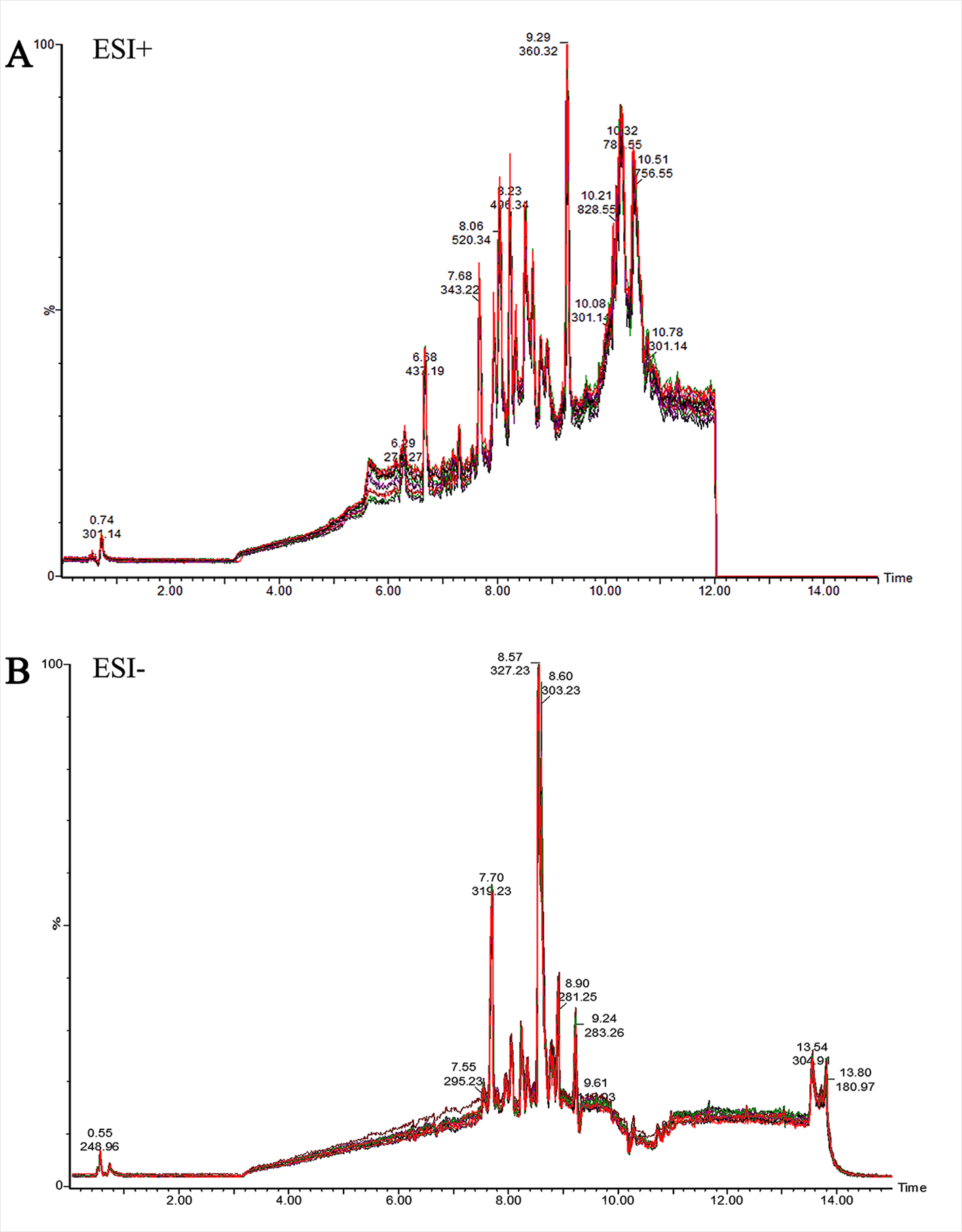

Supplement: Figure S4 (A) — Total ion current (TIC) chromatograms of liver samples in the positive ion mode (ESI+). (B) Total ion current (TIC) chromatograms of liver samples in the negative ion mode (ESI–). [file Image_4.TIF]

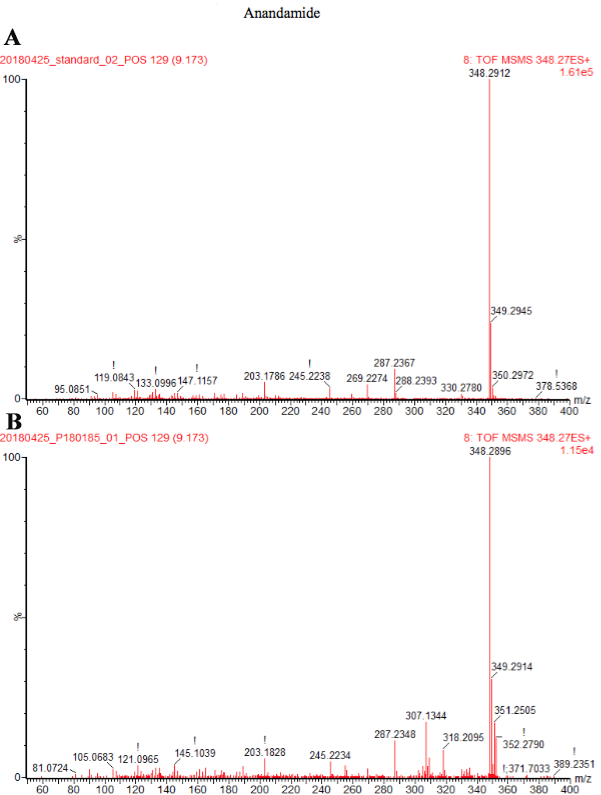

Supplement: Figure S5 — Secondary spectrogram information of anandamide. (A) Standard secondary spectrogram of anandamide in the positive ion mode (ESI+). (B) Secondary spectrogram of experimental samples at similar retention time in the positive ion mode (ESI+). [file Image_5.TIF]

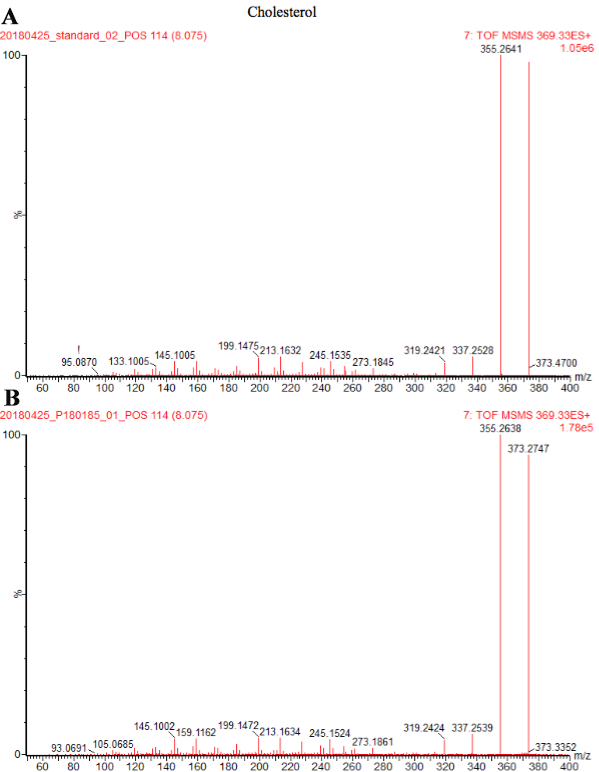

Supplement: Figure S6 — Secondary spectrogram information of cholesterol. (A) Standard secondary spectrogram of cholesterol in the positive ion mode (ESI+). (B) Secondary spectrogram of experimental samples at similar retention time in the positive ion mode (ESI+). [file Image_6.TIF]

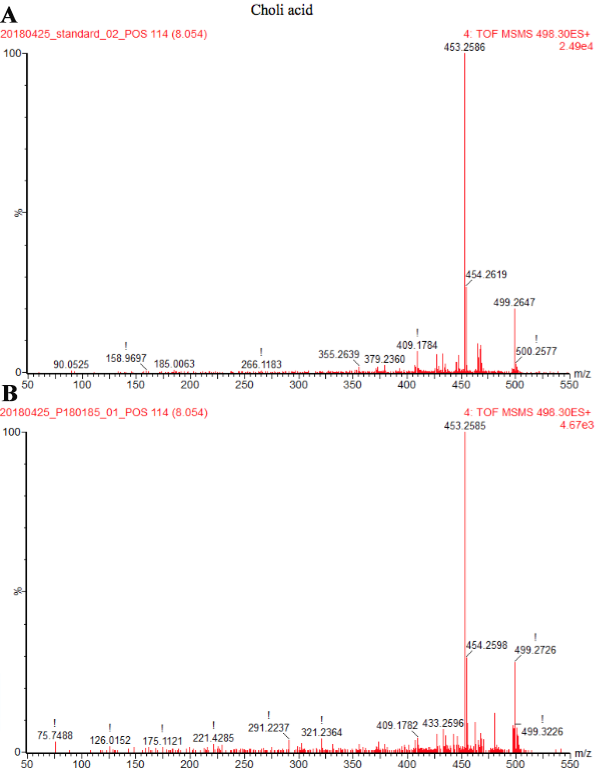

Supplement: Figure S7 — Secondary spectrogram information of choli acid. (A) Standard secondary spectrogram of choli acid in the positive ion mode (ESI+). (B) Secondary spectrogram of experimental samples at similar retention time in the positive ion mode (ESI+). [file Image_7.TIF]

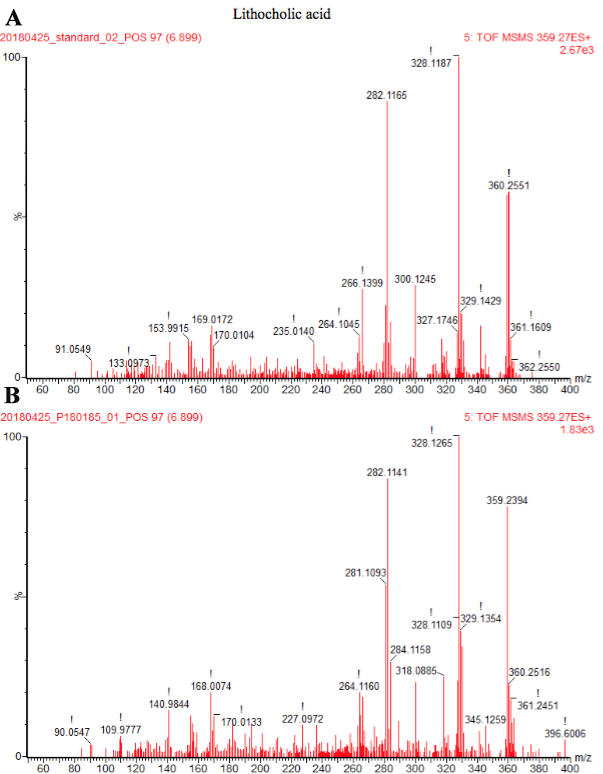

Supplement: Figure S8 — Secondary spectrogram information of lithocholic acid. (A) Standard secondary spectrogram of lithocholic acid in the positive ion mode (ESI+). (B) Secondary spectrogram of experimental samples at similar retention time in the positive ion mode (ESI+). [file Image_8.TIF]

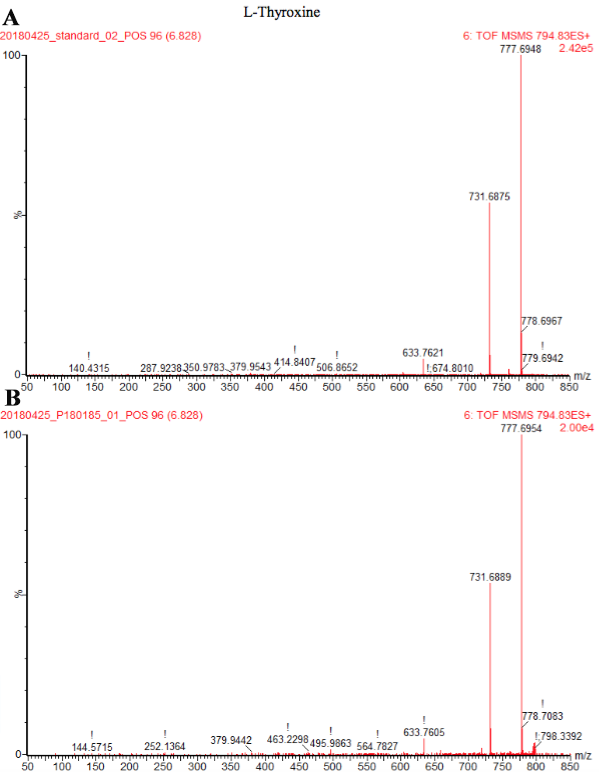

Supplement: Figure S9 — Secondary spectrogram information of L-Thyroxine. (A) Standard secondary spectrogram of L-Thyroxine in the positive ion mode (ESI+). (B) Secondary spectrogram of experimental samples at similar retention time in the positive ion mode (ESI+). [file Image_9.TIF]

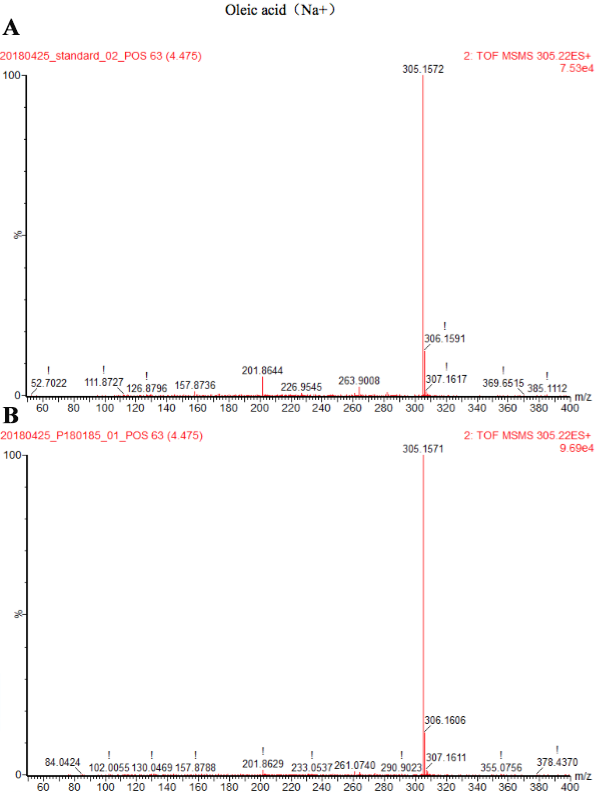

Supplement: Figure S10 — Secondary spectrogram information of oleic acid. (A) Standard secondary spectrogram of oleic acid in the positive ion mode (ESI+). (B) Secondary spectrogram of experimental samples at similar retention time in the positive ion mode (ESI+). [file Image_10.TIF]

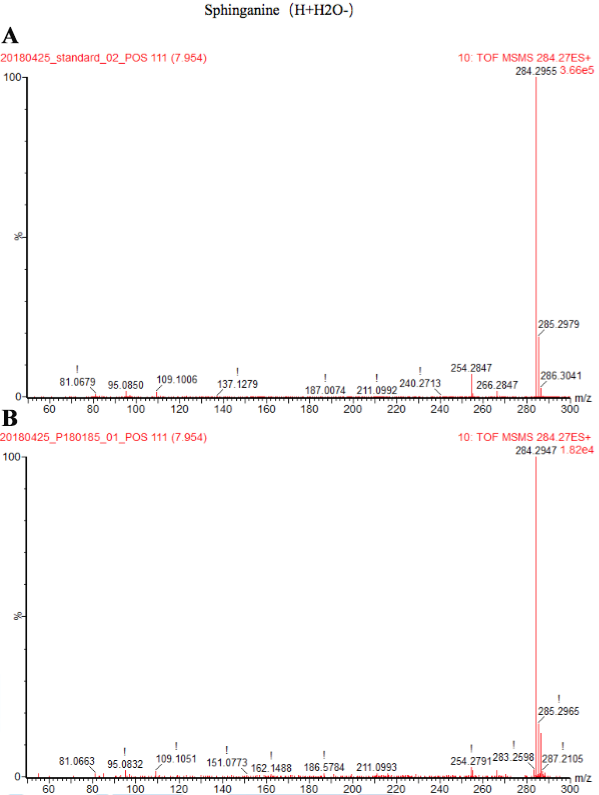

Supplement: Figure S11 — Secondary spectrogram information of sphinganine. (A) Standard secondary spectrogram of sphinganine in the positive ion mode (ESI+). (B) Secondary spectrogram of experimental samples at similar retention time in the positive ion mode (ESI+). [file Image_11.TIF]
