# Supplementary material for: Hepatic Metabolomics Investigation in Acute and Chronic Murine Toxoplasmosis
Source: Front Cell Infect Microbiol. 2018 Jun 5;8:189. doi: 10.3389/fcimb.2018.00189 (PMC5996072; doi:10.3389/fcimb.2018.00189)
Supplement: Table S1 — Summary results of the differential ions. [file Table_1.DOCX]

| Ionization method | Mice group | Differential ion number | Up (MS) | Down (MS) | Up (MS_2_) | Down (MS_2_) |
| --- | --- | --- | --- | --- | --- | --- |
| ESI+ | AI vs CON | 867 | 657 | 210 | 252 | 152 |
|  | CI vs CON | 785 | 602 | 183 | 248 | 146 |
| ESI- | AI vs CON | 898 | 696 | 202 | 186 | 116 |
|  | CI vs CON | 767 | 598 | 169 | 165 | 99 |

**Additional file 2: Table S1** Summary results of the differential ions

**^*^** AI, CI and Con indicate acutely infected, chronically infected and control mouse groups, respectively; MS refers to first mass spectrometry; MS_2_ refers to secondary mass spectrometry.

**^†^**  Up and down indicate up-regulated and down-regulated differential ion number, respectively .
